# Trophic upgrading and mobilization of wax esters in microzooplankton

**DOI:** 10.7717/peerj.7549

**Published:** 2019-08-19

**Authors:** Keyana Roohani, Brad A. Haubrich, Kai-Lou Yue, Nigel D’Souza, Amanda Montalbano, Tatiana Rynearson, Susanne Menden-Deuer, Christopher W. Reid

**Affiliations:** 1 Science and Technology, Bryant University, Smithfield, RI, USA; 2 Chemistry, University of Nevada, Reno, Reno, NV, USA; 3 Graduate School of Oceanography, University of Rhode Island, Narragansett, RI, USA; 4 Marine Science Institute, University of California, Santa Barbara, Santa Barbara, CA, USA

**Keywords:** *Oxyrrhis marina*, Wax ester, Resource deprivation, Trophic upgrading, Catabolism, Microzooplankton

## Abstract

Heterotrophic protists play pivotal roles in aquatic ecosystems by transferring matter and energy, including lipids, from primary producers to higher trophic predators. Using *Oxyrrhis marina* as a model organism, changes to the non-saponifiable protist lipids were investigated under satiation and starvation conditions. During active feeding on the alga *Cryptomonas* sp., the *O. marina* hexane soluble non-saponifiable fraction lipid profile reflected its food source with the observed presence of long chain mono-unsaturated fatty alcohols up to C25:1. Evidence of trophic upgrading in *O. marina* was observed with long chain mono-unsaturated fatty alcohol accumulation of up to C35:1. To the best of our knowledge, this is the first evidence that heterotrophic dinoflagellates are capable of producing ester derived alcohols and that dinoflagellates like *O. marina* are capable of synthesizing fatty alcohols up to C_35_. Additionally, we show evidence of trophic upgrading of lipids. During a 20-day resource deprivation, the lipid profile remained constant. During starvation, the mobilization of wax esters as energy stores was observed with long chain fatty alcohols mobilized first. Changes in lipid class profile and utilization of wax esters in *O. marina* provides insight into the types of lipids available for energy demand, the transfer of lipids through the base of marine food webs, and the catabolic response induced by resource deprivation.

## Introduction

Heterotrophic dinoflagellates are ubiquitous, important components of the pelagic protozoan community. They are significant consumers of bacterial and phytoplankton biomass, and contribute to the cycling of organic matter and nutrients, serving as important trophic links in marine microbial food webs (Strom, 1991; Sherr & Sherr, 1994; Steinberg & Landry, 2017). Trophic interactions within complex marine food webs can strongly influence pathways and efficiencies of material and energy transfer to higher level consumers (Anderson & Menden-Deuer, 2017; Mitra & Flynn, 2005; Rose et al., 2011). Heterotrophic protists, like dinoflagellates, add biochemical value during this transfer through the production and chemical elaboration of compounds (Klein Breteler et al., 1999). Thus changes to the diet of heterotrophic dinoflagellates (i.e., through starvation) can alter the biomass and cellular composition of herbivores. Although heterotrophic protists add biochemical value during trophic transfer, little is known about how cellular composition changes in response to food availability. These changes in biomass can impact higher trophic levels through changes in cellular composition. It has been shown that heterotrophic dinoflagellates such as *Oxyhrris marina*, *Gyrodinium dominans*, and *G. spirale* can survive long periods (>10 days) without algal prey. For example, starvation of *O. marina* for up to 3 weeks resulted in a reduction in cell volume of 17–57% with some cells deformed and transparent (Menden-Deuer et al., 2005). It has been puzzling how a single celled heterotrophic organism can sustain survival in the absence of substantive organic matter, particularly over such extended periods.

Lipids are important energy stores that can be used in times of resource deprivation. Many of the studies on lipids of dinoflagellates fed on algal prey have focused on fatty acid and sterol composition (Klein Breteler et al., 1999; Veloza, Chu & Tang, 2006; Park et al., 2016). These studies have suggested that the fatty acid composition of *O. marina* may not be dependent on its prey and have highlighted this organism’s ability to upgrade lipids acquired from its diet. A subclass of the neutral lipids, wax esters, have traditionally only been found in marine animal phyla (Sargent, Gatten & McIntosh, 1977; Bauermeister & Sargent, 1979), but some examples have been reported in zooplankton species. Wax esters have been observed in the chlorophyte *Chlorella kessleri* (Sargent, Gatten & Henderson, 1981), the cryptomonad *Chroomonas salina* (Antia Naval et al., 1974; Henderson & Sargent, 1989), and the euglenoid *Euglena gracilis* (Furuhashi et al., 2015). In *Chroomonas salina* ester derived alcohols are almost exclusively saturated with the most predominant species C_13_ and C_15_ while in *E. gracilis* ester alcohol moieties of up to C_22_ have been observed.

The mechanism of trophic upgrading by heterotrophic protists may bridge the gap and deliver essential nutrients between higher trophic levels (Klein Breteler et al., 1999; Veloza, Chu & Tang, 2006). Given the importance of heterotrophic dinoflagellates in marine food webs by providing essential nutrients to higher trophic levels, an understanding of the changes to the lipid profile under varying availability of prey can provide insight into the nutritional quality available to higher trophic levels. Here, we report the changes to the non-saponifiable fraction (NSF) lipid composition of the representative dinoflagellate *O. marina*, as our model organisms during active feeding and in response to long term starvation. *O. marina* is a free living, cosmopolitan, and phagotrophic alveolate that feeds on a variety of algae and bacteria (Landry et al., 2000) and has been recognized for its unique starvation ability lasting for several months (Menden-Deuer et al., 2005; Calbet et al., 2013).

## Materials and Methods

### Materials

Long chain fatty alcohol standards were obtained from Millipore Sigma (Burlington, MA, USA). High pressure liquid chromatography (HPLC) lipid standards included a phospholipid mixture and mono-, di-, and tri-acylglycerol mixtures (Millipore Sigma, Burlington, MA, USA). Nile Red was purchased from Invitrogen (Carlsbad, CA, USA). All solvents used were of HPLC or spectroscopic grade. All HPLC mobile phases were filtered through a 0.22 μm membrane prior to use.

### Cell culture

Non-axenic cultures of the cryptophyte alga *Cryptomonas* sp. were maintained in triplicate two-L, transparent polycarbonate (PC) bottles to serve as prey under culture conditions that included a 12:12 h light-dark cycle at 14 °C, salinity of approximately 30 practical salinity units (PSU), and a light intensity of 70–80 µmol photons · m^−2^ · s^−1^. The culture medium was prepared from sterile autoclaved 0.2 µm filtered seawater amended with nutrients following the f/2 medium without silica recipe of Guillard (1975). The seawater was collected at high tide from Narragansett Bay, Rhode Island, USA.

Non-axenic, clonal cultures of the heterotrophic dinoflagellate *O. marina* (CCMP3375; Om), were established via single-cell isolation and grown in two-L transparent PC bottles on a 12 h:12 h light–dark cycle at 14.5 °C, salinity of approximately 30 psu, and a light intensity of 8–15 µmol photons · m^−2^ · s^−1^. Grazers were maintained in exponential growth phase by feeding them once a week with *Cryptomonas* sp. prey and diluted with autoclaved filtered seawater.

### Estimating cell abundance, size, and biomass

Grazer and phytoplankton prey abundance and cell size were monitored using a Multisizer TM 3 Coulter counter (version 3.53; Beckman Coulter, Indianapolis, IN, USA). The Coulter counter provided a more rapid and reliable sampling approach than microscopy and allowed convenient monitoring of the cultures over the course of the experiments (Kim & Menden-Deuer, 2013). Grazer and phytoplankton prey were easily distinguishable on the Coulter counter based on their respective size distributions. Grazer volume was determined using the equivalent spherical diameter measurements from the Coulter counter, and converted to carbon biomass (pg C. cell^−1^) using previously established conversion equations (Menden-Deuer & Lessard, 2000).

Starvation and re-feeding experiments were set-up using established methods (Anderson & Menden-Deuer, 2017). Briefly, grazers fed with prey were transferred to triplicate four-L bottles and starved for 1–3 weeks until a reduction in predator abundance or cell size was detected, which indicated a negative impact of algal prey deprivation and marked the initiation of grazer starvation (Anderson & Menden-Deuer, 2017). Grazers were starved for 20 days, received a fresh pulse of phytoplankton prey after starvation, and were monitored for 3–7 days after re-feeding. Samples were taken at regular intervals (0, 8, 10, 15, 18, 20 days) during the starvation for measurements of grazer abundance and cell size. Grazer and prey abundances obtained using the coulter counter and flow cytometer were verified by light microscopy.

### Lipid isolation

Filter membranes containing *Cryptomonas* sp. or *O. marina* cells were extracted using the Bligh-Dyer procedure (Bligh & Dyer, 1959). Briefly, 10^5^ to 10^7^ cells adhered to membrane were suspended in five mL of 1:2 (v/v) CHCl_3_:MeOH and sonicated for 5 min at 65% amplitude, (10 s on, 20 s off). Lipids were extracted with the addition of three mL of 2:1 MeOH:CHCl_3_ and mixed by vortexing. The sample was then converted to a two phase Bligh-Dyer by the addition of one mL CHCl_3_ and 1.8 mL dH_2_O. The resulting biphasic samples were centrifuged (4,000 rpm, 2 min) and the bottom layer was recovered and transferred to a clean sample vial. The organic layer was dried under a stream on N_2_. Samples were then analyzed by HPLC. Portions of the lipid extracts were subjected to saponification (methanolic KOH) (Haubrich et al., 2015) for analysis of neutral lipids and analyzed by gas chromatography-mass spectrometry (GCMS) without further derivatization. Negative controls of solvent extracted filter membranes were incorporated into the experiment to control for contaminants arising from the filter membranes.

### Lipid staining and flow cytometry

Samples were stained with Nile Red as described by De la Jara et al. (2003). The optimal fluorescence of Nile Red can be highly selective and variable based on dye concentration and cell type stained (Rumin et al., 2015), and given the novelty of Nile Red staining with marine heterotrophic dinoflagellates, we followed an optimization protocol. The effect of several parameters on dye permeation and fluorescence were tested such as final dye concentration (0.5–5 µg mL^−1^), incubation time (5–30 min), solvents (e.g., DMSO and acetone), and temperature (Rumin et al., 2015). An optimum Nile Red concentration of two µg mL^−1^ dissolved in acetone was determined based on fluorescence profiles (via flow cytometry), and thus represented the dye concentration used in starvation experiments. Triplicate five mL samples were spiked with Nile Red, gently vortexed, and incubated for 10 min at room temperature in the dark to ensure dye permeation while avoiding quenching effects. Nile Red samples were then fixed using glutaraldehyde (0.5% final conc. v/v), flash frozen in liquid N_2_, and stored at −80 °C until flow cytometry analysis (measured within 1–2 months).

Triplicate samples were analyzed using flow cytometry (BD-Influx flow cytometer, Becton Dickinson Instruments) with an excitation wavelength of 488 nm. A minimum of 200 cells were counted for each sample. Populations of cells were identified based on fluorescence vs forward and side scatter. Chlorophyll autofluorescence was determined using a 692/40 nm filter and differentiated autotrophic prey from grazer lipid fluorescence. Lipid content, measured as fluorescence intensity per cell, was estimated from the fluorescence emission of NR-stained cells using 580/30 nm (neutral lipid), and 610/20 nm (polar lipid) filters (Alonzo & Mayzaud, 1999; De la Jara et al., 2003). Non-stained samples were used to control for NR autofluorescence; fluorescence of unstained cells was consistently less than 10% of stained samples. Lipid content for each grazer species was measured as a function of fluorescence, and expressed as relative fluorescence units, rather than as equivalent lipid concentrations.

### Non-saponifiable lipid extracts

Lipid extracts were treated with 10:10:80 (v/w/v) of dH_2_O:KOH:MeOH and refluxed for 30 min (Haubrich et al., 2015). After cooling to room temperature, water and hexanes were added. Saponified lipids were extracted three times with hexanes and pooled. The combined hexane extracts were dried over anhydrous Na_2_SO_4_ and evaporated to dryness under a stream of N_2_. Prior to analysis samples were dissolved in equal volumes of CHCl_3_.

### Chromatographic analysis

#### RP-HPLC

Chromatographic separation was performed on a Prominence uHPLC system (Shimadzu, Columbia, MD, USA) equipped with a COSMOSIL 5 μ C18-MS II (4.6 × 150 mm) column (Nacalai Tesque Inc., Kyoto, Japan). Lipid classes were separated employing a binary gradient system described by Knittelfelder et al. (2014) with detection at 205 nm (Guarrasi et al., 2010). Mobile phase A consisted of water:methanol (1:1 v/v), mobile phase B was 2-propanol. Both solvents contained phosphoric acid (eight μM) and formic acid (0.1% v/v). A linear gradient with initial conditions starting at 45% mobile phase B was increased to 90% B over 30 min. Mobile phase B was then increased to 100% over 2 min and was held at 100% for 10 min. The column was re-equilibrated for 15 min between injections. Retention time regions for polar, and neutral lipids were established using commercial lipid standards. Data was analyzed using LabSolutions (Shimadzu, Columbia, MD, USA) and statistical analysis (ANOVA) performed using GraphPad Prism version 7.0. All samples were run in biological triplicate and technical duplicate.

#### GCMS

NSF lipid samples were analyzed GCMS with an Agilent 7890A gas chromatograph equipped with a 5975C electron impact mass spectrometer set to 70 eV using a Restek Rtx-5 column (30 m × 25 μm diameter). The GC flow rate of He was set at 1.2 mL/min, injector port set to 250 °C, and the initial temperature set at 170 °C, held for 1 min, then increased at 20 °C/min to a final temperature of 280 °C (Haubrich et al., 2015). Chromatograms were processed using ChemStation (Agilent, Santa Clara, CA, USA) and analyte identification of the resulting chromatograms was performed via interrogation of resulting electron impact mass spectra with the NIST database and manual analysis. Octadecanol (RT = 5.001 min) was the standard for determination of relative retention time. All samples were run in biological triplicate and technical duplicate.

## Results and discussion

### *O. marina* lipid class profile

**Figure 1:**
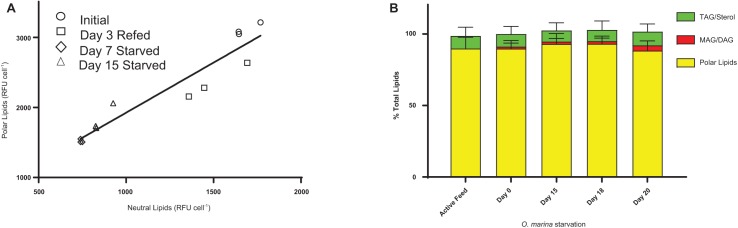
*O. marina* maintains a constant ratio of polar:neutral lipids during a 20-day starvation period. (A) Linear relationship between depletion of polar and neutral lipids during starvation measured using flow cytometry. *O. marina* feeding on *Cryptomonas* sp. polar vs neutral lipids is characterized by a significant, linear relationship (model II regression, *p* < 0.0001, *r*^2^ = 0.903). (B) RP-HPLC analysis of lipid class. *O. marina* were fed *Cryptomonas* sp. prior to start of starvation. *O. marina* maintains a balance of 92.1 ± 3.2:3.0 ± 2.2:5.7 ± 2.6 (phospholipid (PL):monoacyl/diacylglyerols (MAG/DAG):triacylglycerol/sterol/wax ester (TAG/sterol/WE) as cell volume decreases. Lipid extracts were analyzed in biological triplicate and technical duplicate. Statistical analysis (ANOVA, *p* < 0.01) of lipid class abundance indicates no significant difference during the starvation period.

The lipidome of protist predators can change rapidly in response to environmental conditions. Nile Red lipid staining of *O. marina* demonstrated significant changes in the concentration of neutral and polar lipids in satiated and starved cells (Fig. 1A). A linear relationship (*r*^2^ = 0.9044) in the decrease in polar and neutral lipids during starvation was observed via flow cytometry (Fig. 1A). The apparent differences in total polar and neutral lipids after 7 and 15 days respectively are non-significant (*p* > 0.05). The change in polar and neutral lipid concentration during starvation was further investigated by RP-HPLC. While total polar and neutral lipids decreased during starvation the relative abundance of the subclasses of these lipids showed a consistent ratio of polar to neutral lipids of 92.1 ± 3.2:3.0 ± 2.2:5.7 ± 2.6 (PL:MAG/DAG:TAG) was maintained (Fig. 1B). The lipid class composition of *O. marina* is comparable to what has been previously observed in dinoflagellates and phytoplankton (Harvey et al., 1988; Bourdier & Amblard, 1989; Yoon et al., 2017). Nutrient deprived *O. marina* have been shown to decrease cell volume by 17–57% (Anderson & Menden-Deuer, 2017) and increase expression of genes involved in the degradation of lipids (Rubin et al., 2019) suggesting a homeostatic requirement for *O. marina* to maintain relative amounts of each lipid class as cell volume decreases and stress-induced catabolism progresses.

### GCMS analysis of hexane soluble non-saponifiable fraction

**Figure 2:**
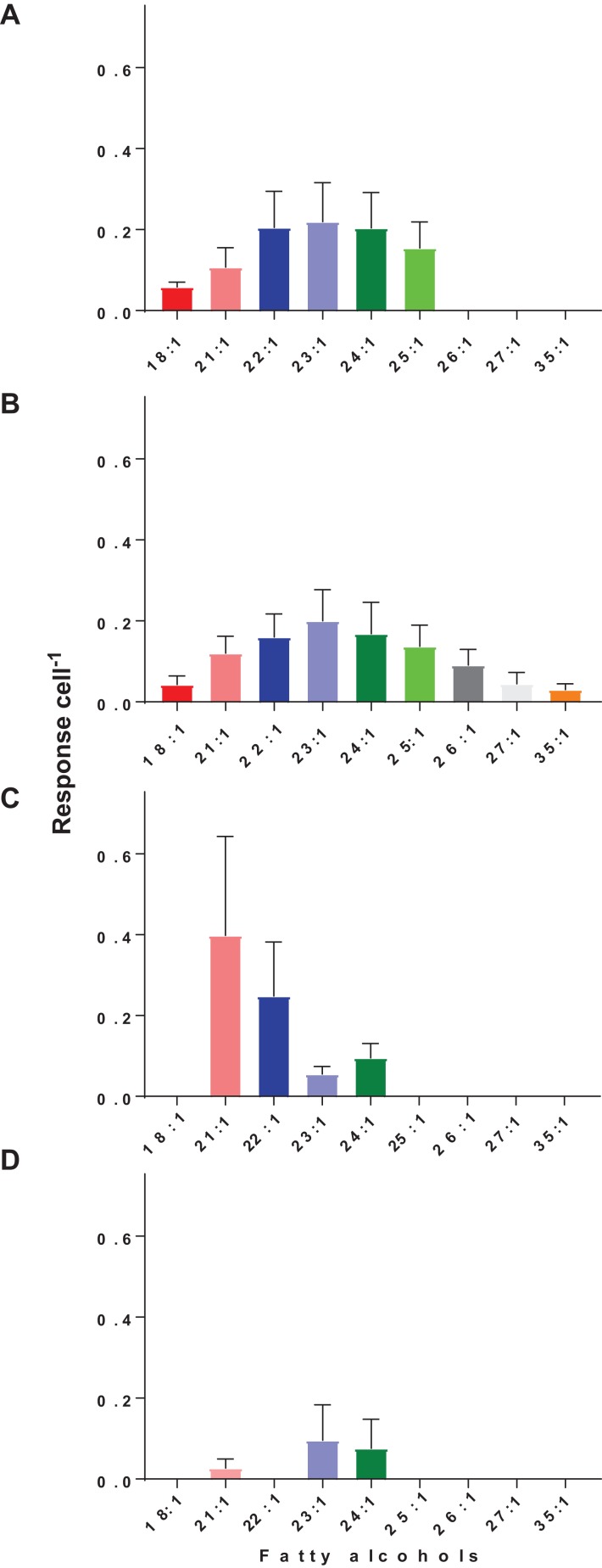
Changes in hexane soluble NSF lipid extracts during active feeding and prolonged starvation of *O. marina*. (A) Prey *Cryptomonas* sp., (B) *O. marina* during active feeding on *Cryptomonas* sp., (C) *O. marina* after 15 days starvation, (D) *O. marina* after 18 days starvation. Day zero of starvation commenced when prey were not detectable by Coulter Counter and microscopy. Evidence of trophic upgrading of observed fatty alcohols in actively feeding *O. marina*. During a 20-day starvation, *O. marina* mobilized wax esters as an energy source.

The prey *Cryptomonas* sp. contained wax ester-derived mono-unsaturated fatty alcohols ranging in size from C18:1 to C25:1 (Fig. 2A; Fig. S1; Table S1). These results are in contrast to observations in other phytoplankton in which fatty alcohols up to C_22_ have been observed (Henderson & Sargent, 1989; Furuhashi et al., 2015). Our results obtained here for *Cryptomonas* sp. indicate the presence of both even and odd chain alcohols.

*Oxyrrhis marina* actively feeding on *Cryptomonas* sp. showed the same base fatty alcohol profile as its prey. In addition to the *Cryptomonas* sp. derived fatty alcohols, there was evidence of trophic upgrading in that derived fatty alcohols were upgraded to chain lengths of up to C_35_, which were not detected in the prey fatty alcohol profiles (Fig. 2B). To the best of our knowledge, this is the first evidence that heterotrophic dinoflagellates are capable of producing ester derived alcohols and that dinoflagellates like *O. marina* are capable of synthesizing fatty alcohols up to C_35_, compared to zooplankton species from arctic waters where mono-unsaturated fatty alcohols up to C_22_ have been observed (Sargent, Gatten & McIntosh, 1977; Wakeham, 1982).

Over a 20 day starvation period, *O. marina* appeared to mobilize the fatty alcohols as energy reserves with longer chain fatty alcohols utilized first (Figs. 2C and 2D). After 20 days near complete depletion of fatty alcohols was observed, consistent with observations of increased expression of genes involved in lipid degradation in starved *O. marina* (Rubin et al., 2019).

Wax ester production in dinoflagellates has been suggested to be involved in buoyancy regulation and as a deposit of an energy rich food reserve during periods of low prey abundance (Sargent, Gatten & Henderson, 1981). Wax esters and TAGs are commonly found in lipid bodies within dinoflagellates. These compounds have been the focus of investigations during nitrogen stress (Dagenais Bellefeuille et al., 2014) and in coral-dinoflagellate symbiont relationships (Chen et al., 2012). This analysis of changes in lipid class profile and utilization of wax esters in *O. marina* provides insight into the catabolic response induced by general resource deprivation. We have provided evidence that during starvation in *O. marina* that wax esters are mobilized as energy stores while the ratio of polar:non-polar lipids remain constant as cell volume decreases. These data provide information on the changes in lipid content, in particular the NSF in *O. marina* during prolonged resource deprivation.

## Conclusions

Here, we evaluated the lipid profile of a marine herbivorous zooplankton during starvation and contrasted this with the NSF lipid profile of its phytoplankton prey. We found evidence both of direct trophic transfer of lipids from the algal source, as well as trophic upgrade of neutral lipids. While diet deprivation did not seem to affect ratios of polar to neutral lipids in starved *O. marina*, starvation was accompanied by a time-dependent depletion of longer-chain fatty alcohols from energy stores. In addition, the presence of remarkably long fatty alcohols was noted in saponified lipids of a heterotrophic dinoflagellate. Characterization and quantification of catabolic responses to resource stress in marine herbivores provides opportunities to use lipids as biomarkers for energy demand and assessment of energy status in marine microbial food webs and improve coastal ecosystem models.

## Supplemental Information

10.7717/peerj.7549/supp-1 Supplemental Information 1 Identified non-steryl alcohol species in the prey (*Cryptomonas* sp.) and the model dinoflagellate *O. marina* during starvation from Fig. S1. Compounds were identified through NIST library search and manual analysis of associated EIMS spectra.

10.7717/peerj.7549/supp-2 Supplemental Information 2 Representative GCMS chromatograms of hexane-soluble NSF lipid extracts. (A) *Cryptomonas* sp. and (B) *O. marina* during active feeding and (C) 15 and (D) 20 days of starvation, respectively. Day zero of starvation commenced when prey were not detectable by Coulter counter and microscopy. During a 20 day starvation, *O. marina* mobilized wax esters as energy source.

10.7717/peerj.7549/supp-3 Supplemental Information 3 Chromatographic (GCMS, HPLC) and fluorescence raw data for lipid analysis of Cryptomonas sp. and *O. marina* during active feeding and starvation. Each file chromatographic file (GCMS, HPLC) has been converted to the corresponding Analytical Data Interchange format (.cdf). Flow cytometry raw data is provided as an excel spreadsheet.

## Additional Information and Declarations

### Data Availability

The following information was supplied regarding data availability: Raw data in the format of GCMS and HPLC chromatograms are provided as .netCDF files in the Supplemental Materials.
