# Supplementary material for: Trophic upgrading and mobilization of wax esters in microzooplankton
Source: PeerJ. 2019 Aug 19;7:e7549. doi: 10.7717/peerj.7549 (PMC6705382; doi:10.7717/peerj.7549)
Supplement: Supplemental Information 1 — Compounds were identified through NIST library search and manual analysis of associated EIMS spectra. [file peerj-07-7549-s001.docx]

| **Retention Time (min)** | **RRT^a^** | **M^+^** | **Assigned Alcohol** | **Chromatogram Peak Number^b^** |
| --- | --- | --- | --- | --- |
| 5.01 | 1.00 | 268 | 18:1 | 1 |
| 6.18 | 1.27 | 310 | 21:1 | 2 |
| 6.57 | 1.35 | 324 | 22:1 | 3 |
| 6.97 | 1.43 | 338 | 23:1 | 4 |
| 7.39 | 1.52 | 352 | 24:1 | 5 |
| 7.86 | 1.61 | 366 | 25:1 | 6 |
| 8.40 | 1.67 | 380 | 26:1 | 7 |
| 9.01 | 1.79 | 394 | 27:1 | 8 |
| 9.74 | 1.94 | 506 | 35:1 | 9 |

1. RRT relative retention time (reference octadecanol 5.001 min)
2. Refer to labeled peaks in Figure 1.
