# Supplementary material for: Trophic upgrading and mobilization of wax esters in microzooplankton
Source: PeerJ. 2019 Aug 19;7:e7549. doi: 10.7717/peerj.7549 (PMC6705382; doi:10.7717/peerj.7549)
Supplement: Supplemental Information 2 — (A) Cryptomonas sp. and (B) O. marina during active feeding and (C) 15 and (D) 20 days of starvation, respectively. Day zero of starvation commenced when prey were not detectable by Coulter counter and microscopy. During a 20 day starvation, O. marina mobilized wax esters as energy source. [file peerj-07-7549-s002.pdf]

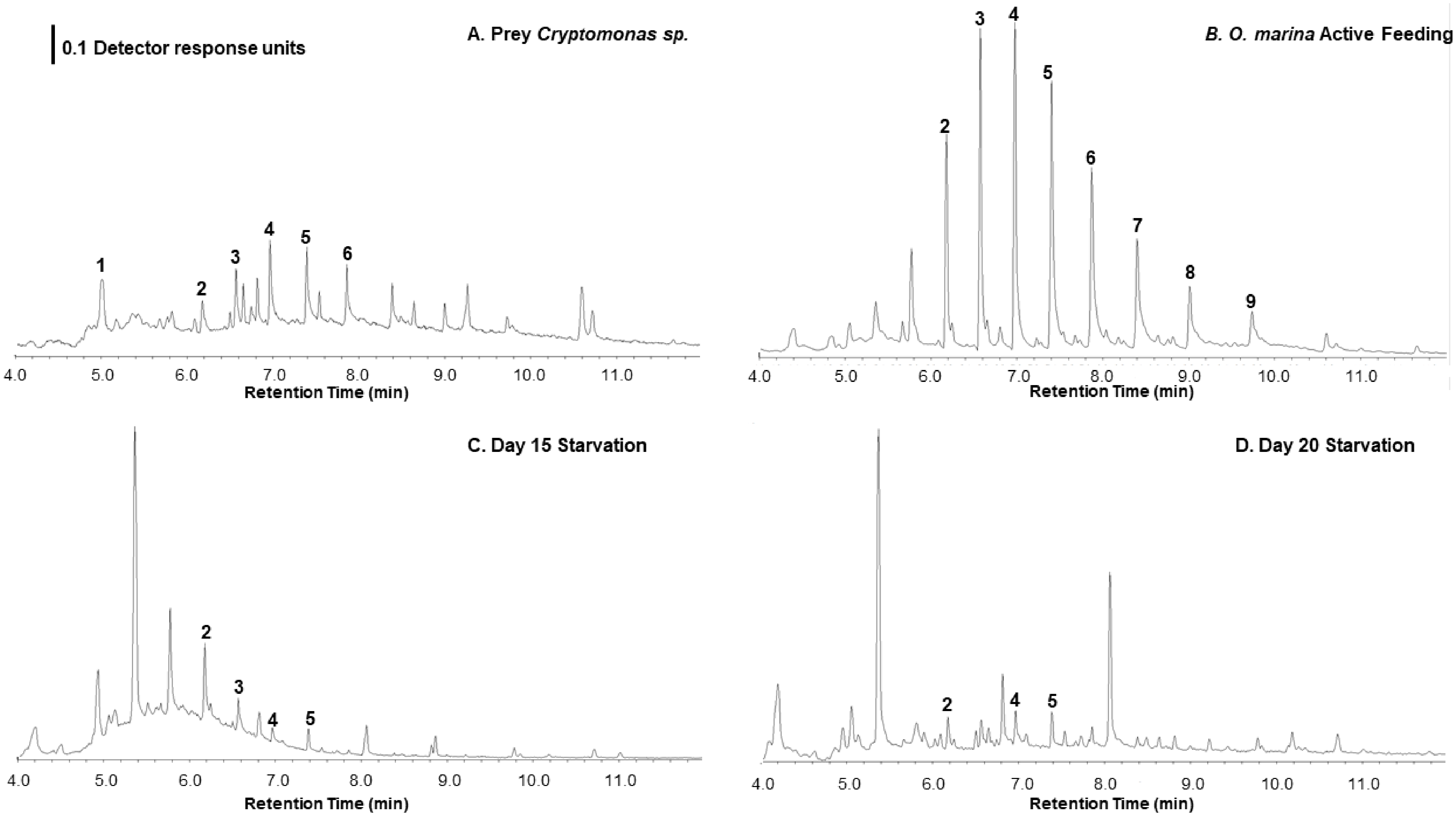
0.1 Detector response units
A. Prey *Cryptomonas sp.*
B. O. marina Active Feeding
C. Day 15 Starvation
D. Day 20 Starvation
1
2
3
4
5
6
7
8
9
4.0
5.0
6.0
7.0
8.0
9.0
10.0
11.0
Retention Time (min)
